# Cellular senescence and cancer: Focusing on traditional Chinese medicine and natural products

**DOI:** 10.1111/cpr.12894

**Published:** 2020-09-03

**Authors:** Yiman Liu, Shenshen Yang, Kailong Wang, Jia Lu, Xiaomei Bao, Rui Wang, Yuling Qiu, Tao Wang, Haiyang Yu

**Affiliations:** ^1^ Tianjin State Key Laboratory of Modern Chinese Medicine Tianjin University of Traditional Chinese Medicine Tianjin China; ^2^ School of Pharmacy Tianjin Medical University Tianjin China

**Keywords:** active ingredients, cell senescence, Traditional Chinese Medicine, tumour

## Abstract

Cancer is the principal cause of death and a dominant public health problem which seriously threatening human life. Among various ways to treat cancer, traditional Chinese medicine (TCM) and natural products have outstanding anti‐cancer effects with their unique advantages of high efficiency and minimal side effects. Cell senescence is a physiological process of cell growth stagnation triggered by stress, which is an important line of defence against tumour development. In recent years, active ingredients of TCM and natural products, as an interesting research hotspot, can induce cell senescence to suppress the occurrence and development of tumours, by inhibiting telomerase activity, triggering DNA damage, inducing SASP, and activating or inactivating oncogenes. In this paper, the recent research progress on the main compounds derived from TCM and natural products that play anti‐cancer roles by inducing cell senescence is systematically reviewed, aiming to provide a reference for the clinical treatment of pro‐senescent cancer.

## Graphical abstract

As an important anti‐tumour function, cell senescence could prevent the cellular replication of damaged DNA. Cell senescence is usually stimulated by damages, such as telomere shortening, DNA damage and oncogene‐induced senescence. Active ingredients of TCM and natural products, can induce cell senescence to suppress the occurrence and development of tumours, through inhibiting telomerase activity, triggering DNA damage, inducing SASP, and activating or inactivating oncogene.

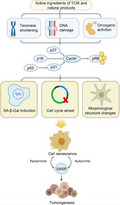

## 1 INTRODUCTION

### 1.1 Malignancy and current treatment

The incidence of malignant tumours is rising worldwide. Cancer is regarded as the leading cause of death and the most key obstacle to improve life expectancy in every country of the world in the 21st century.^1^ Tumour is a new organism formed by the proliferation of local tissue cells under the action of various tumorigenic factors. The development of cancer consists of a number of complex stages of initiation, progression and promotion, in which the stage of progression is invertible which seems to be the phase for the most appropriate drug intervention. Although there are different types therapies targeting cancers, such as surgical resection, radiotherapy, chemotherapy, immunotherapy, biotherapy therapy, molecular‐targeted therapy and treatment of TCM, however, not every therapy achieves the expected optimal effect results. For instance, surgical resection can remove the tumour, the risk of advanced cancer is increased, and the survival rate is reduced. Clinical combination of first‐line chemotherapy drugs and radiotherapy can prevent the progress of cancer to some extent, but they may cause serious toxic effects, affect the metabolism and proliferation of normal tissues, and reduce the life quality of patients. Furthermore, the limitation of radiotherapy is that it can cause hair loss, memory damage and even lead to a second cancer. The function of organs, such as brain, muscle and bone, will decline in the late radiotherapy. In addition, secondary cancer and normal tissue damage resulting from chemotherapy also bring clinical problems for the cancer survivor. In the course of chemotherapy, most chemotherapy drugs can cause bone marrow suppression, resulting in immune deficiency or decline. Some chemotherapy drugs can also cause liver toxicity, kidney toxicity, cardiac toxicity and so on. Even the low sensitivity of some cancer cells means that chemotherapy has little or no effect on overall survival, which may have a significant impact on prognosis. For immunotherapy, it is important to avoid autoimmune diseases during tumour treatment. Patients who benefit from these treatments cannot be predicted due to unclear therapeutic targets and mechanisms. Biological therapy and molecular‐targeted therapy usually have little effect on middle and advanced cancer patients in most cases, and the therapeutic effect is not satisfactory. Currently, both treatments are prone to tumour recurrence and metastasis.

So far, there is no widely accepted optimal treatment option for cancer treatment. Hence, it is necessary to find out new treatment options and more effective treatment methods to treat malignant tumours, especially specific targeted drug therapies. Data from the WHO report that the most of global population living in developing countries rely on traditional medicines extracted from plants for primary health care.^2^ Recently, studies have shown that many TCM and monomer compounds, such as flavonoids, phenylpropanoids and alkaloids, have significant anti‐tumour effects.^3^, ^4^, ^5^ Nowadays, TCM has been used for cancer care and acts as an active function in healthcare system due to their low toxicity, strong specificity and high efficacy. As an important source of active natural products, TCM has the characteristics of multi‐component and multi‐target, which could not only restrict the tumorigenesis, but also reduce the side effects of radiotherapy and chemotherapy. In addition, it also shows unique advantages in the tumour prevention and treatment, especially in enhancing the host immune function and prolonging the survival of patients.^6^ At present, in clinical practice, TCM is often used to assist the application of chemotherapy drugs in order to enhance the efficacy of chemotherapy drugs and improve the quality of life of patients.

### 1.2 Cell senescence and anti‐tumour

Cell senescence is an irreversible cell cycle arrest state in which senescent cells permanently lose their capacity to proliferate. Senescent cells are characterized by morphological and metabolic changes, chromatin remodelling, gene expression changes and the emergence of a pro‐inflammatory phenotype known as senescence‐associated secretion phenotype (SASP). Cell senescence plays an important role in the prevention of cardiovascular, kidney, liver and other diseases, as well as tissue regeneration and repair, anti‐tumour and other aspects.^7^ Currently, cell senescence identifies as innate barrier in the formation of tumour and induces senescent growth stagnation of tumour cells, which has been a novel idea in anti‐tumour research. Interestingly, previous studies have shown the role of cell senescence in promoting cell death against tumour. Cyclin‐dependent kinase 4 and 6 (CDK4/6) is a pivotal protein for cell cycle regulation, especially in phase G1 the mutation of which is closely related to the occurrence of tumour. According to the report, in the mouse models of KRAS mutant pancreatic ductal adenocarcinoma(PDAC), the combination of trametinib (MEK inhibitor) and palbociclib (CDK4/6 inhibitor) can induce PDAC cell senescence. The senescence‐inducing treatment produces a SASP that includes angiogenic factors that promote tumour angiogenesis, thereby improving the efficacy of drug delivery and cytotoxic gemcitabine chemotherapy.^8^ Specific E3 ubiquitin ligase, MDM2, can bind with wild‐type p53 protein and inactivate it, that is, MDM2‐mediated degradation of p53. Nutlin‐3a, an MDM2 antagonist, can inhibit the degradation of p53, which enables p53 to play its role in inducing the senescence of leukaemia cells. Interestingly, the Nutlin‐3a induced senescence of cells is irreversible.^9^ Therefore, to balance the function between cell vitality and cell proliferation, inducing cancer cell senescence is a promising therapeutic model.

In addition to inhibiting the vitality and metastasis of cancer cells, regulating autophagy and apoptosis, blocking the cell cycle, active ingredients of TCM and natural medicine can also play an anti‐tumour role by promoting cell senescence. The senescence process induced by TCM and natural drugs is relatively slow compared with other anti‐tumour strategies, whereas a welcome benefit of it is that it does not cause widespread damage to surrounding tissues or skin. Therefore, the active components of TCM or natural drugs can induce the tumour cells senescence and selectively eliminate senescent cells, which can reduce inflammation and enhance the function of the immune system, thus delaying the progress of age‐related diseases, improving health and prolonging life. The elderly cells activate the proliferative mechanisms of various organs to fight against cell senescence together, so that the cancer cells in the body have the opportunity to develop into tumours. As a consequence, selective scavenging of senescent cells is of great significance to prevent the occurrence and development of tumours. The anti‐tumour effect of TCM through this mode tends to be in the treatment of middle and advanced cancer, and the long‐term effect of which is much better. The outstanding advantage of TCM is to restore the balance in the body. For this reason, the paper summarizes the research status in regulating cell senescence of anti‐tumour effects through TCM and natural products, in order to provide reference for the development of advanced cancer therapies by combining TCM with modern cutting‐edge technologies.

## 2 REGULATION OF CELLULAR SENESCENCE BY ACTIVE COMPOUNDS DERIVES FROM TCM

**FIGURE 1:**
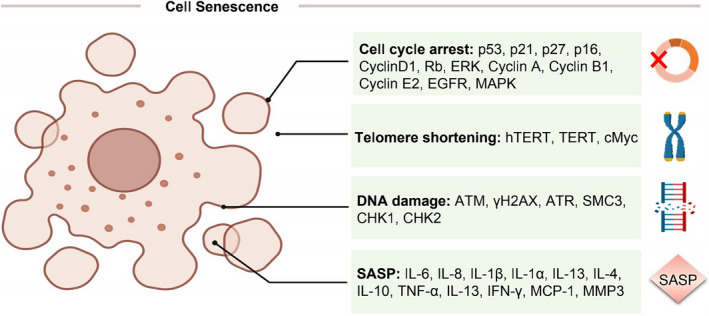
The main biological phenomenon and related proteins that regulate cell senescence

Senescence is usually triggered by damaging stimuli, and cellular senescence can be divided into replicative senescence and stress‐induced premature senescence.^10^ In this section, the detailed mechanisms of TCM regulating senescence in cancer cells will be described. Meanwhile, the main biological phenomena and related proteins are roughly summarized for reference (Figure 1).

### 2.1 Inhibition of telomerase activity induces cell senescence

**FIGURE 2:**
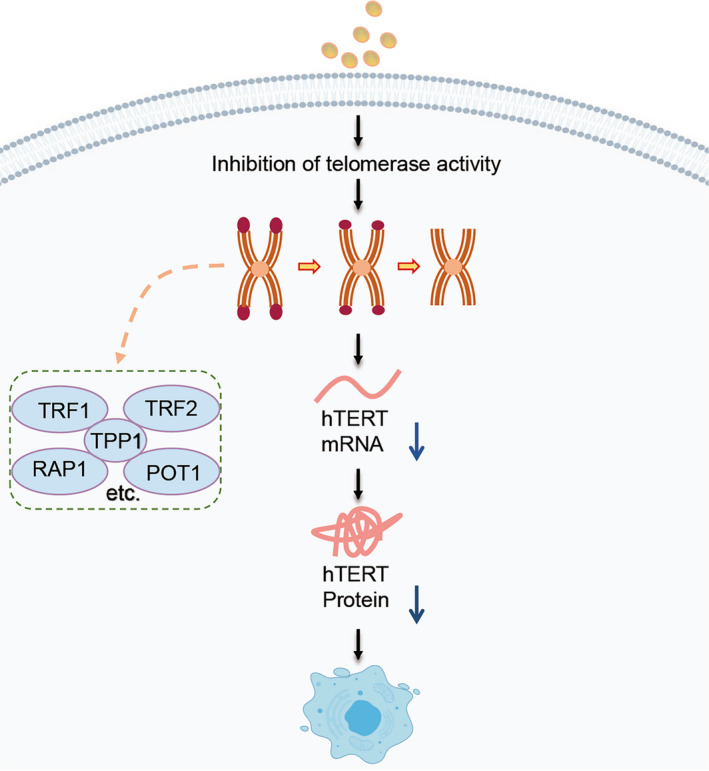
The main mechanism of telomerase activity regulates cell senescence

Normal non‐mutant cells after undergoing 50 divisions (called Hayflick limit) will stop dividing, which is known as replicative senescence. Replication senescence is generally considered as one of the anti‐cancer mechanisms of eukaryotes, which is caused by the shortening of telomeres during replication. During each round of DNA replication, telomeres gradually shorten, eventually reaching a critical length so that it could prevent further replication and thus stops the cell from division. Shorter and capped telomeres induce a DNA damage response, thereby triggering senescence. Telomere length is maintained by telomerase, which is expressed in the large majority of cancers cells.^11^, ^12^ Telomerase activity is insufficient to maintain balance with the rapid rate of cell proliferation, resulting in telomere shortening and cell ageing.^13^ Tumour cells tend to increase telomere length and promote ageing‐related genes mutations by activating telomerase so as to live on forever.^14^ As a consequence, pre‐senescence and tumour cells death can be induced by inhibiting telomerase activity and preventing telomere prolongation.^11^, ^14^ It has become one of the most promising strategies for cancer therapy to inhibit tumour generation via suppressing telomerase activity to induce cell senescence (Figure 2).

#### 2.1.1 Phenolics

Ginger (*Zingiber officinale* Roscoe), the fresh roots of the perennial herbs of the ginger family, has a long history of medicinal plant study. It also has been reported to exhibit various biological activities, such as antimicrobial, anti‐tumour, anti‐inflammatory activity and resistance to diabetes.^15^, ^16^ Kaewtunjai et al found that subcytotoxic doses of *Z officinale* extract (ZOE) could cause telomere shortening and trigger cell senescence in A549 lung cancer cells. Moreover, active compounds in ZOE (6‐paradol, 6‐shogaol) were determined, which may be responsible for the suppression of hTERT expression and telomerase activity. The result also revealed that ZOE had no acute toxicity in rats, and along with anticlastogenic effect against liver micronucleus formation. In conclusion, ZOE had been shown to inhibit telomerase activity and subsequently induce cell senescence, thereby demonstrating its remarkable anti‐tumour property.^17^

Pterostilbene (PT, a dimethyl ether analog of resveratrol) exhibits semblable pharmacological bioactivities. Compared with resveratrol, PT has better pharmacokinetic properties.^18^ It has been indicated that an excellent interaction occurred between PT and the active site of telomerase.^19^ Rong‐Jane Chen et al^20^ discovered that PT‐induced senescence partly emerged via a p53‐dependent mechanism as follows: PT → suppression of telomerase activity → DNA damage → ATM/ATR/p53 activation → p21 induction → blocked S phase → cellular senescence.

#### 2.1.2 Flavonoids

Baicalein, a phenolic flavonoid compound, which is an active ingredient of *Scutellaria baicalensis* Georgi, has been reported to possess various bioactivities^21^, ^22^ Baicalin, the conjugate of baicalein (baicalein‐7‐glucuronide), also exists in *S baicalensis*.^23^ Cumulative evidence shows that both baicalin and baicalein exert powerful anti‐cancer effects in various cancers.^24^ Jie Dou et al revealed that treatment with baicalein and baicalin could significantly hinder tumorigenesis in vivo and vitro. Induction of senescence has been shown to be a possible mechanism involved in suppressive effect in colon cancer cells. Comparing hTERT expression treated with baicalin or baicalein, baicalin dramatically downregulated hTERT expression. In addition, both baicalin and baicalein could significantly induce cell senescence in vivo. Besides, MAPK ERK and p38 signalling pathways, related to the regulation of colon cancer cell senescence, were selectively mediated by baicalein and baicalin.^25^

#### 2.1.3 Terpenoids

Triptolide (TPL) is a typical diterpenoid isolated from Chinese medicinal herb *Tripterygium wilfordii* Hook F.^26^ Researchers have suggested that TPL processes extensive activities including anti‐tumour, anti‐fertility and anti‐cystogenesis effects.^27^ The results have revealed that TPL could obviously increase p53 and p21^Kip1^, proving that TPL could block cell cycle. In short, TPL promoted cell senescence and inhibited the growth of liver cancer by inhibiting cyclin and activating AKT signalling pathway. In addition, TPL could suppress the expression of hTERT and telomerase activity, contributing to acceleration of cell senescence which resulted in inhibition of tumour growth.^28^

#### 2.1.4 Alkaloids

Paclitaxel, as a natural diterpenoid, was exacted from the bark and trunk of Yew Pacific. Known as the best natural anti‐cancer drug that has been discovered, it restrains depolymerization and improves the stability of tubulin ultimately contributes to inhibit the growth of tumour cells.^29^, ^30^ Multani et al studied and contrasted the effects of paclitaxel with its water‐soluble complexes on chromosome morphology and apoptosis induction in K1735 clone X‐21. The results showed that paclitaxel and its water‐soluble conjugates could inhibit the occurrence and development of tumour by inducing extensive telomere erosion. These compounds also could stimulate chromosomal fusion and telomerase dysfunction.^31^ Park et al^32^ demonstrated that, upon telomere erosion, paclitaxel propagated chromosomal instability via stimulating chromosomal end‐to‐end fusions and delaying the development of multinucleation by use of telomerase‐deficient cells derived from mTREC‐/‐p53‐/‐ mice.

Papaverine, well known as a type of isoquinoline alkaloid, is derived from *Papaverine somnifrum* L,^33^ which has been shown to possess a unique and wide bioactivity for clinical use including smoothing muscle relaxant, gastrointestinal dysfunction and cough.^34^ Based on previous researches, Sakineh Kazemi Noureini et al demonstrated that papaverine could inhibit telomerase by downregulating hTERT in spite of interacting indirectly with telomeric sequences and dramatically reduce transcription level of hTERT, which is closely related to the inhibition of telomerase activity. On the other hand, they also found that prolonged treatment with low doses of papaverine could accelerate the senescence of HepG2cell and β‐Galactosidase staining verified it.^35^ These results well explained that papaverine could restrain the development of HCC by inhibiting telomerase activity and inducing cell senescence.

Harmine isolated from the seeds of *Peganum harmala* L. and *Banisteriopsis caapi*, which belongs to the natural β‐carboline alkaloid. As a folk medicine, harmine shows various remarkable pharmacological effects, such as antimutagenic, antidepressant and antiplatelet.^36^ Lei Zhao et al indicated that harmine arrested cell proliferation and induced cellular senescence in MCF‐7 cells, confirmed by positive SA‐β‐gal staining result. In the research, according to the telomeric repeat amplication protocol assay, harmine induced a notable decrease of telomerase activity in MCF‐7 cells, which was accompanied by a significant downregulation of all the hTERT subunits.^37^ To sum up, harmine has a prominent effect on regulating cell senescence which is accompanied by a significant suppression of telomerase activity via overexpressed factor of p53/p21 pathway.

#### 2.1.5 TCM extracts

Tianshengyuan‐1 (TSY‐1) is exacted via a distillation process of multiple Chinese herbs, involving mangnolia officinalis, schisandra Chinensis, pericarpium citri reticulatae viride and almond. It has been reported that TSY‐1, an agent used to treat bone marrow deficiencies, has an outstanding effect stimulating telomerase activity.^38^, ^39^ Weibo Yu et al discovered that TSY‐1 suppressed telomerase activity in HL60 cells but increased telomerase activity in PBMCs and HSCs. The result confirmed that TSY‐1 has a reverse effect on leukaemia cells with inherently high telomerase activities (telomerase inhibitory) and normal blood mononuclear and stem cells with low telomerase activities (telomerase activator). It seemed that the effect of TSY‐1 on telomerase activity in different cell systems may be mediated by the methylation of the TERT promoter. Furthermore, the β‐galactosidase reporter staining assay also revealed that the effect of TSY‐1 on telomerase activity in connection with cell senescence.^40^ Taken together, TSY‐1 exhibited an effect on maintaining telomerase homoeostasis of telomerase activity for telomerase‐based target treatment of bone marrow deficiency and cancer.

Telomerase is highly overexpressed in 85% of tumour but is almost undetectable in most normal tissues. Therefore, it is suitable to be an anti‐cancer target. As mentioned above, a large number of researches have indicated that various native compounds could reduce telomerase activity and hTERT mRNA/protein levels. Another approach used to inhibit telomerase activity is to target telomere. In this case, inhibition of telomere extension is a potential anti‐cancer therapeutic strategy. To some extent, we can develop telomerase inhibitors based on TCM. It should be noted that there are still many serious problems to be solved, such as which stage the telomerase activation will occur, whether anti‐telomerase treatment will affect the immune response of the body, and when the active ingredients of TCM will inhibit telomerase activity so as to promote cell senescence and block tumour growth.

### 2.2 DNA damage and cellular senescence

**FIGURE 3:**
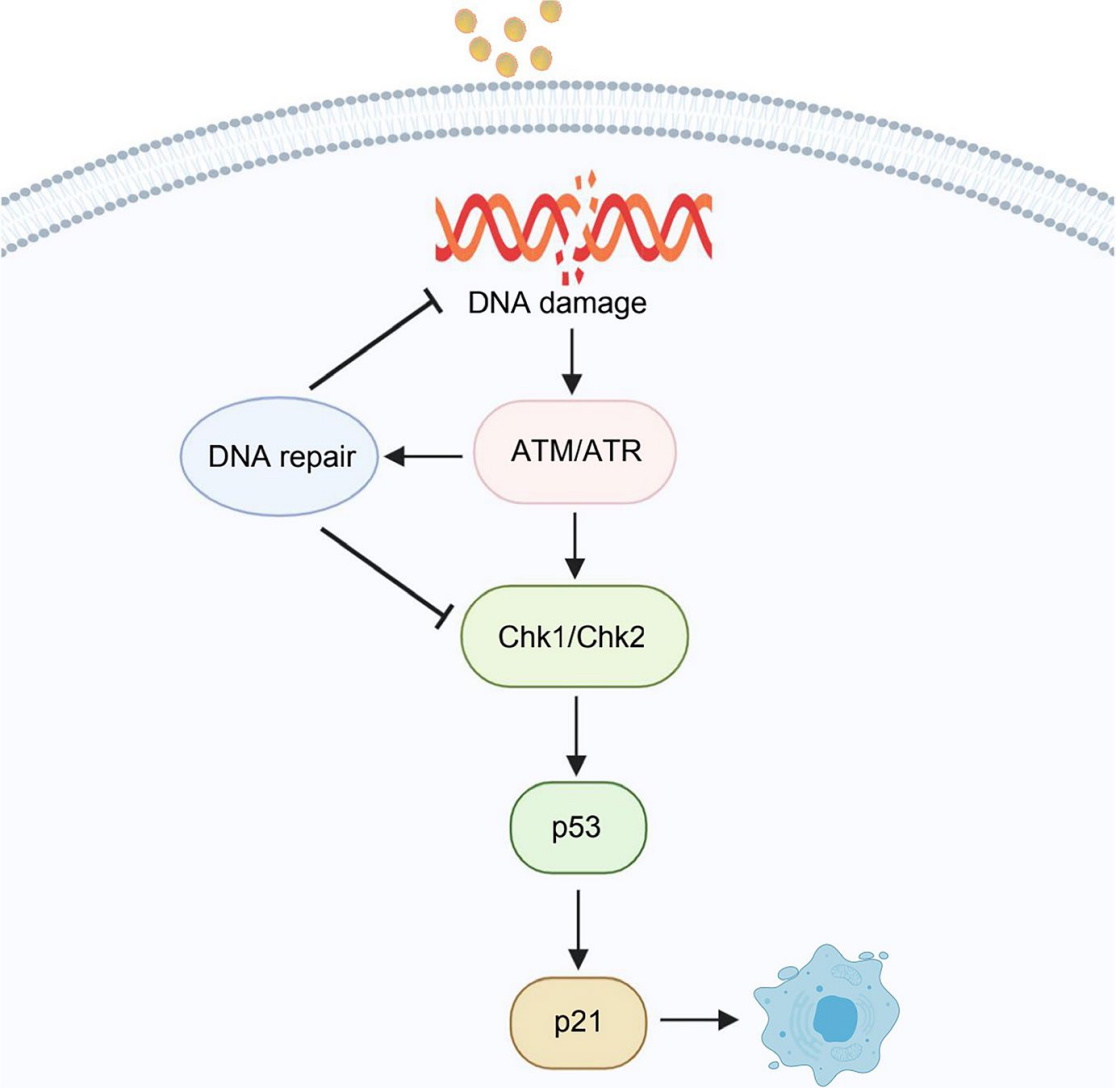
The relationship of DNA damage and cell senescence

DNA damage, as another inducement of cell senescence, can trigger DNA repair mechanisms, apoptosis or senescence, on the basis of the damage degree and the physiological environment. Senescent cells are characterized by sustained DNA damage response (DDR), including chronic ataxia‐telangiectasia mutated gene (ATM) and the ATM‐ and Rad3‐related (ATR) kinases signal transduction, which eventually induces cell cycle arrest stagnation and senescence by activating the p53/p21 and p16/pRb pathways.^41^ Sustained DNA damage and subsequent senescence can also be induced by ionizing radiation, chemotherapy, genotoxic stress and oxidative stress. However, erroneous DNA repair may lead to chromosomal aberrations and mutations, which drive carcinogenesis in case of affecting oncogenes and tumour suppressor genes. Alternatively, unrepaired DNA lesions can cause cell dysfunction or senescence and eventually cell death (Figure 3). More importantly, DNA damage not only damages the regeneration ability of stem cells but also destroys the integrity of tissues, ultimately leading to various pathologies during the process of aging.^42^

Icaritin (ICT) is a prenylflavonoid derivative extracted from *Epimedium brevicornu* Maxim. (Berberidaceae).^43^ Previous studies have shown that ICT could attract increased attention for its inhibition of various tumours.^44^ Shikang Wang et al discovered that treatment with low doses of ICT induced cell senescence in HCC cells, as observed by enlarged and flattened morphology and increased β‐galactosidase activity, along with cell cycle arrest at G0/G1 phase and reduction of DNA synthesis. Furthermore, ICT enhanced the expression level of γH2AX (a well‐known indictor in response to DNA damage), in accordance with the immunofluorescence results. These results revealed that low concentration of ICT induced ROS generation and subsequently stimulated ROS‐mediated DDR.^45^

Berberine is derived from *Berberis* genus plants and belongs to isoquinoline alkaloids, has been used in Chinese folk medicine for thousands of years.^46^ Numerous studies found that berberine has diverse properties of anti‐inflammation, antioxidation, hepatoprotective and antiarrhythmic properties. Besides, berberine inhibits cell viability against various cancer cells, accompanied by its low toxicity in normal cells.^47^ Yun‐Xia Xiong confirmed that the suppression of cancer cells vitality by Ber8 was connected with cell cycle arrest, cell senescence and DNA damage at telomere regions. Ber8 caused DNA damage along with the increase of p‐ATM, p‐p53 and γH2AX. In conclusion, berberine could stabilize telometric endogenous G‐quadruplexes and cause DNA damage in the telomere region and telomere end uncaping, thereby inducing cellular senescence in cancer cells.^48^

Hinokitiol, a natural monoterpenid isolated from the essential oil of *Calocedrus formosana* heartwood, is a safe and bioactive phytochemical with a wide range of molecular targets and exerts numerous biological activities involving remarkable anti‐cancer property.^49^ Lan‐Hui Li et al demonstrated that the expression of LC3‐II, p62 and ATG5, recognized as established markers of autophagosome formation, increased after treatment with hinokitiol. Besides, Hinokitiol elevated levels of γH2AX and unchanged level of p53. More importantly, hinokitiol induced cell cycle arrest in S phase and triggered cell senescence to prevent cell replication in lung adenocarcinoma cells. Hinokitiol‐induced senescence was attenuated by contreatment with autophagy inhibitors 3‐MA, which further supported the relevance between autophagy and senescence. Moreover, it was proved that hinokitiol caused DNA damage and autophagy followed by cell cycle arrest and senescence.^50^ Taken together, the results demonstrate that hinokitiol may be a candidate therapeutic agent for the treatment of lung adenocarcinoma.

Acetyl‐11‐keto‐b‐boswellic acid (AKBA), a pentacyclic triterpene acid extracted from the fragrant gum resin of plant *Boswellia serrata* tree, has been widely used clinically for treatment of diverse inflammatory diseases.^51^, ^52^ Shikang Wang et al proved that low concentration of AKBA induced premature senescence in HCC, as supported by arresting increase in β‐galactosidase activity and DNA synthesis. AKBA‐induced cell senescence occurred by activating DNA damage response accompanied by impairment of DNA repair. It was proved that AKBA treatment induced γH2AX and p53, and downregulated expression of DNA repair‐related genes, such as RAP1, MCM2‐7 and PCNA. Furthermore, induction of p53 by AKBA resulted in a remarkable increase of p21^CIP1^, which is believed to be closely related with cell senescence. Therefore, AKBA induced senescence of HCC via causing DNA damage response accompanied by impairment of DNA repair genes, which is conducive to the anti‐cancer function of AKBA.^53^

The plant secondary metabolite gallotannin (GT, the polyphenolic hydrolysable tannin), derived from green tea, has been exerted multiple biological roles in anti‐bacterial and anti‐cancer.^54^ Racha Al‐Halabi et al revealed that GT, as a DNA damaging drug, inhibited cell proliferation and DNA synthesis, and induced S‐phase arrest in HCT116 cell. Interestingly, low dosages of GT‐induced senescence were evidenced by an increase in β‐galactosidase stained cells and the appearance of SAHFs, which was independent of cellular status of p21 and p53 with the partial involvement of ROS in this effect. In addition, DNA damage induced by GT was demonstrated by enhanced immunofluorescence and increased γH2AX expression.^55^ Their results suggest that GT has potential value as an anti‐cancer agent for the treatment of colon cancer.

Curcumin, an ancient natural substance derived from *Curcuma longa*, plays an important role in the prevention and treatment of cancer.^56^ Previous studies have proved that cancer cells treated with curcumin show mitotic disruption and induce cell senescent stagnation. It was subsequently demonstrated that the mitotic spindle had formed improperly during mitosis, resulting in the upregulation of γH2AX expression as well as the increase in expression of p53 and p21. In addition, treatment with curcumin leads to double‐stranded DNA damage and activates the DNA damage response, thus inducing cell senescence. Later, the researchers conducted the experiment using the caffeine (ATM/ATR inhibitor), which turned out that pre‐treatment of cancer cells with caffeine could eliminate the accumulation of curcumin‐induced mitotic cells. In other words, the results also suggest that in the presence of caffeine, curcumin treatment also attenuates cell senescence.^57^

DNA damage is an intermediate process between telomerase and cell senescence induced by tumour stress. Since senescent cells generally have the ability of resisting apoptosis, it is important to actively seek a treatment to convert the senescent tumour cells into apoptosis. The active ingredients of TCM and natural medicine summarized above can regulate cell senescence by inducing DNA damage. Their common feature is that normal DNA damage monitoring and repair network can regulate the entry of severely damaged cells into apoptosis, while on the other hand, they can regulate the naturally ageing tumour cells into senescence. These drugs provide a reference for the study of anti‐ageing drugs and a feasible strategy for the prevention and treatment of tumours.

### 2.3 Oncogene‐induced Senescence

**FIGURE 4:**
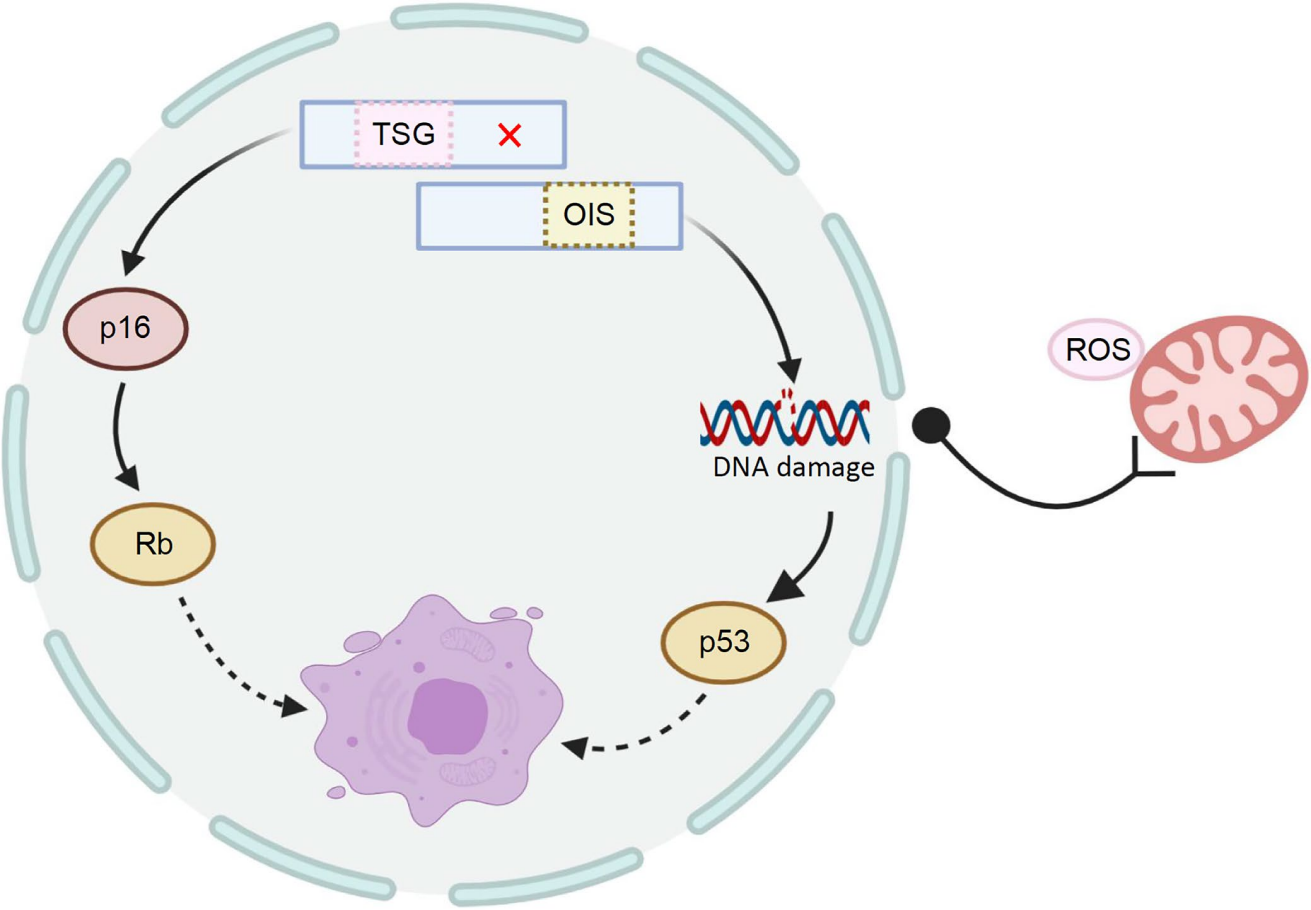
The main mechanism of oncogene‐induced senescence

Senescence can also be triggered by the activation of oncogenes (oncogene‐induced senescence (OIS)) and the loss of tumour suppressor gene (TSG), as a potent cell‐independent anti‐cancer mechanism. Senescence, which occurs in cells undergoing oncogenic signal transduction, is a response to prevent their transformation into malignant cells. It has been scientifically discovered that OIS is typically characterized by the upregulation of cell cycle inhibitors p16^INK4A^ and p21^CIP1^.^58^ During the OIS period, cells undergo a variety of phenotypic changes. For example, chromatin recombination in senescent cells results in the formation of SAHF, which inhibits the growth of senescent cells by silencing proliferation‐promoting genes.^59^ OIS cells require the participation of the p19^ARF^‐p53 and p16‐Rb tumour suppressor pathways, since the inactivation of these tumour suppressor pathways allows cells to bypass oncogenic Ras‐induced senescence (Figure 4).^60^, ^61^

Chansu, a traditional Chinese medicine prepared from the skin and auricular gland secretions of giant toads, has been used not only for treatment of various cardiac diseases, infection and pain for millennium but also for anti‐tumour therapy.^62^ According to previous researches, bufalin exerts anti‐cancer effect by regulating cell differentiation, autophagy and cell cycle.^63^, ^64^ P53 is an important anti‐cancer gene which has been confirmed to participate in many physiological activities of tumour. Yong Zhang et al demonstrated that bufalin induced protein abundance of p53 (not mRNA) and its well‐known transcriptional target p21^CIP1^, as well as G2 phase arrest. Supplementally, the expression of phosphorylation of ATM and γH2AX (Ser139) was increased, revealing that the bufalin's anti‐tumour activity was related to DNA damage. What is more, significant increase of the activity of SA‐β‐gal was observed after bufalin treatment. Furthermore, the results revealed that bufalin‐induced senescence attenuated in case of knocking down of p53, whereas knockout of p21 aggravated bufalin‐induced caspase‐mediated apoptosis.^65^ Their findings suggest that bufalin has potential anti‐cancer effect by activating p53‐senescence in prostate cancer patients. Baicalin extracted from *Scutellaria* root with outstanding biological activities.^66^ Zhou Wang et al indicated that treatment with baicalin observably induced cell senescence in colon cancer cells. Furthermore, DEPP protein expression increased after baicalin treatment in HCT116 cells, accompanied by the activation of Ras/Raf/MEK/ERK and p16/Rb signalling pathways. In addition, overexpression of DEPP could significantly enhance the activity of β‐galactosidase in cancer cells induced by baicalin and vice versa. The results suggest that the powerful effect of DEPP as a key node is participated in the senescent process, providing new insights into other types of cancer treatment.^67^ Avenanthramide A (AVN A) is an active ingredient exclusively extracted from *Avena sativa* L.^68^ Abundant evidences from molecular studies and animal models have shown the efficiency of AVN A for treatment of many diseases due to its anti‐inflammatory, anti‐oxidative and anti‐cancer activities.^69^ According to the enlarged cell size, increased number of blue cells stained with SA‐β‐gal and the positive staining with γH2AX, it was indicated that cell senescence occurred with treatment of AVN A. Significant G1 phase arrest and increase of critical effectors of cellular senescence such as p27, p16 and p21 were observed after AVN A treatment. Moreover, AVN A treatment significantly increased the expression of miR‐129‐3p, which markedly repressed the E3 ubiquitin ligase Pirh2 and two other targets, IGF2BP3 and CDK6. The expression of p53 and p21 was increased after Pirh2 silencing by miR‐129‐3p, suggesting that the anti‐tumour effect of AVN A is related to cell senescence.^70^ The result indicate that AVN A could be a potential agent for colorectal cancer, attributing to the role of miR‐129‐3p/Pirh2/p53 axis in regulation of cellar senescence.

### 2.4 Microenvironment and cellular senescence

**FIGURE 5:**
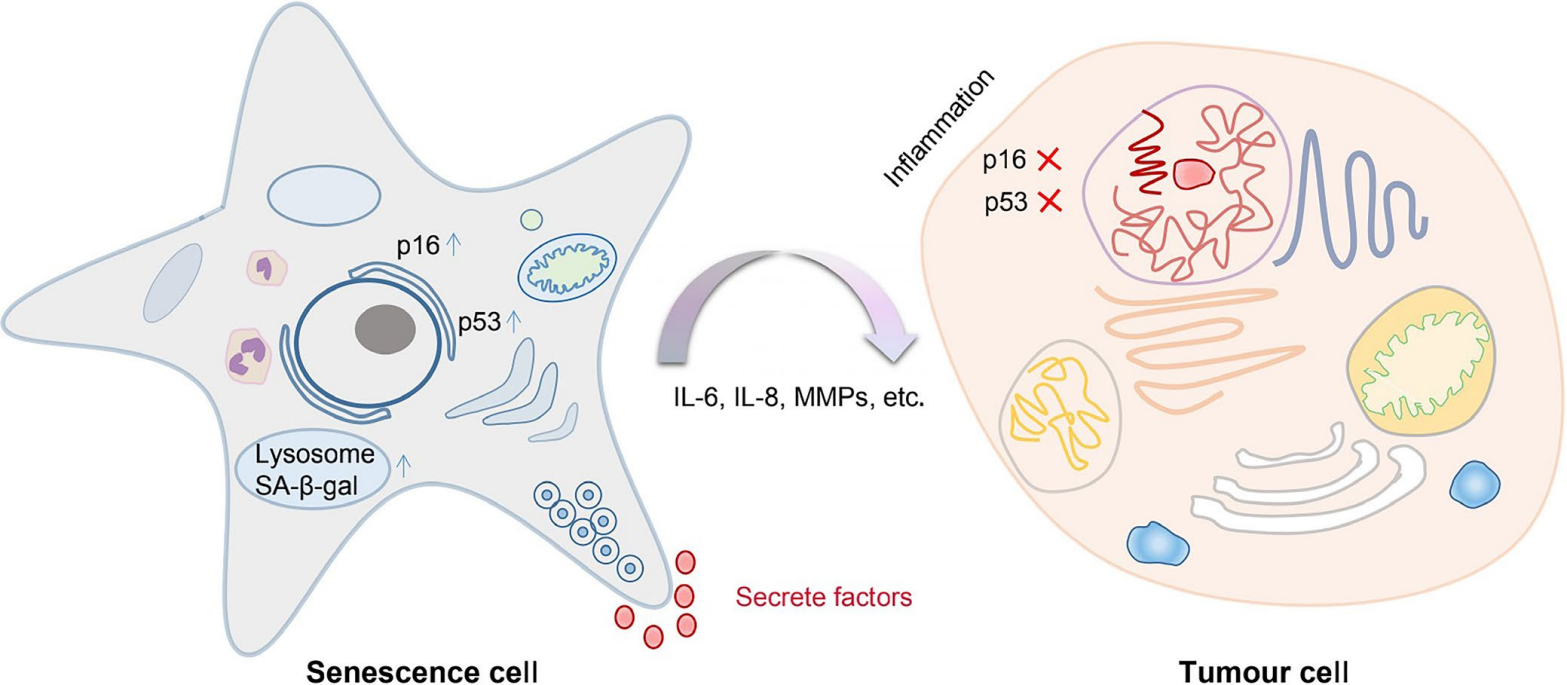
The main mechanism of SASP and cell senescence

One of the characteristics of senescent cells is that they remain metabolically activity to produce and secrete superfluous factors which can affect the tissue microenvironment in different modalities.^71^, ^72^ Many senescent cells exhibit a pro‐inflammatory senescence‐associated secretion phenotype (SASP) that can mediate non‐cellular autonomic senescence effects, whether beneficial or harmful effects. SASP is composed of secreted inflammatory cytokines, proteases, chemokines and growth factors, and contributes to tumour suppression with elimination of senescent cells by triggering immune cells.^73^, ^74^ These secreted factors promote communication with neighbouring cells and the immune system, and ultimately affect the fate of senescent cells. The secreted SASP, especially some pro‐inflammatory cytokines that are tightly regulated by the transcription factor NF‐κB pathway, can recruit immune cells to clear up the damaged cells and premalignant cells to prevent tumorigenesis.^75^ SASP as a communication medium between senescent cells and microenvironment causing the occurrence of ageing and age‐related diseases, such as malignant tumours. Therefore, induction of cellular senescence with minimally increased SASP would be benefit for cancer therapy (Figure 5).

As an active component and natural anti‐cancer substance in pomegranate, punicalagin (PUN) can resist various malignant tumours, including glioma and colon sarcoma.^76^ Xian Cheng et al revealed that PUN treatment induced senescence in papillary thyroid carcinoma BCPAP cells, as observed by increased SA‐β‐gal staining. Cell cycle arrest and upregulation of p21 both further verified cellular senescence induced by PUN. PUN generated SASP in BCPAP cells, which mainly manifested by high levels of inflammatory cytokines, principally IL‐6 and IL‐1β. In order to further explore the relationship between PUN anti‐tumour and SASP, researchers also evaluated the activation of the NF‐κB signalling pathway. It is indicted that phosphorylation and subsequent degradation of IκBα, as well as the nuclear translocation of p65, were caused by PUN exposure. However, in case of inhibition of NF‐κB, the reduction of SA‐β‐Gal staining positive cells was observed, and the SASP generation was blocked.^77^ In sum, PUN treatment induces cell cycle arrest and SASP by the activation of NF‐κB pathway. Marchantin C (Mar‐C) is derived from liverworts *Marchantia polymorpha*.^78^ As a product of natural existence, it has shown a variety of biological activities (eg. anti‐tumour, anti‐fungal), along with low toxicity.^79^, ^80^ Mar‐C treatment could obviously reduce cell viability by inducing cell cycle arrest, increasing the expression of p53, p21 and p27, and decreasing the expression of Rb and pRb. Later, researchers examined the effect of Mar‐C on SASP, and they discovered that upon management with Mar‐C, the secretion of important anti‐inflammatory factors such as IL‐4 and IL‐10 changed dramatically. However, no detailed changes were found in IL8. Taken together, Mar‐C promotes cell senescence with limited cytotoxicity, which acts as a selective SASP modulator, and NF‐κB partly participates in the regulating of SASP, suggesting the promising agent of Mar‐C for lung cancer treatment.^81^

Most anti‐cancer therapies target the cells themselves, but ignore the surrounding microenvironment. In the case of low dose drug administration, senescent cells affect tumour cells by regulating the body's own immune system in the form of autocrine or paracrine. This kind of tumour immunotherapy assisted by TCM will achieve satisfactory results with its advantages of long response time, low adverse reaction and significant anti‐tumour effect, bringing hope to the tumour patients. During the treatment, it can not only control the growth of the tumour, but also improve the immune function from the aspect of remodelling tumour microenvironment regulation.

### 2.5 Other forms

In fact, there are many TCM and natural products that can induce cell senescence to fight tumours. It is not shown in the paper because some active ingredients can induce cell senescence to fight tumour, but the specific mechanisms by which some active ingredients induce senescence are not well understood or not discussed in other ways. For example, 1, 8‐cineole is a typical case. It is the main ingredient of eucalyptus essential oil, which mainly exists in tea, sage and other plants. The researchers revealed that 1, 8‐cineole inhibited cell proliferation by triggering G0/G1 cell arrest followed by cell senescence in HCC HepG2 cell, but not apoptotic. Furthermore, it was indicated that 1, 8‐cineole induced oxidative stress, which was involved in HepG2 cell senescence. Both ROS and cell senescence were responsible for cell growth inhibition mediated by 1, 8‐cineole.^82^ Artemisinin (ART), as its name implies, is derived from the plant *Artemisia annua*. It has been proven that ART has a long medicinal history in China, used for treatment of malaria and cancer.^83^ Jian Chen et al found that ART inhibited the proliferation of lung cancer cells by block the cell cycle. High concentration of ART management promoted the occurrence of apoptosis biological events, whereas low‐concentration ART exposure induced cell senescence. However, the specific mechanism by which ART regulates cellular senescence against cancers is rarely reported in literature.^84^ Gypenoside L (Gyp‐L) is a common active ingredient in Chinese medicine, identified from gynostemma pentaphyllum. Jingxin Ma discovered that Gyp‐L restrained cell growth by inducing cell senescence. In both cases, the researchers revealed that Gyp‐L further promoted cell senescence by activating MAPK and NF‐κB signals that blocked the cell cycle. However, there is no original opinion on the inhibition of telomerase and DNA damage.^85^ Magnolin is the most effective ingredient in the herb *Magnolia fargesii*, which has been proved to have a variety of pharmacological functions. Magnolin could inhibit cell proliferation and transformation by directly targeting ERK1 and ERK2 and inhibiting the ERKs/RSK2 signalling pathways by blocking G1/S cell cycle transduction.^86^

**FIGURE 6:**
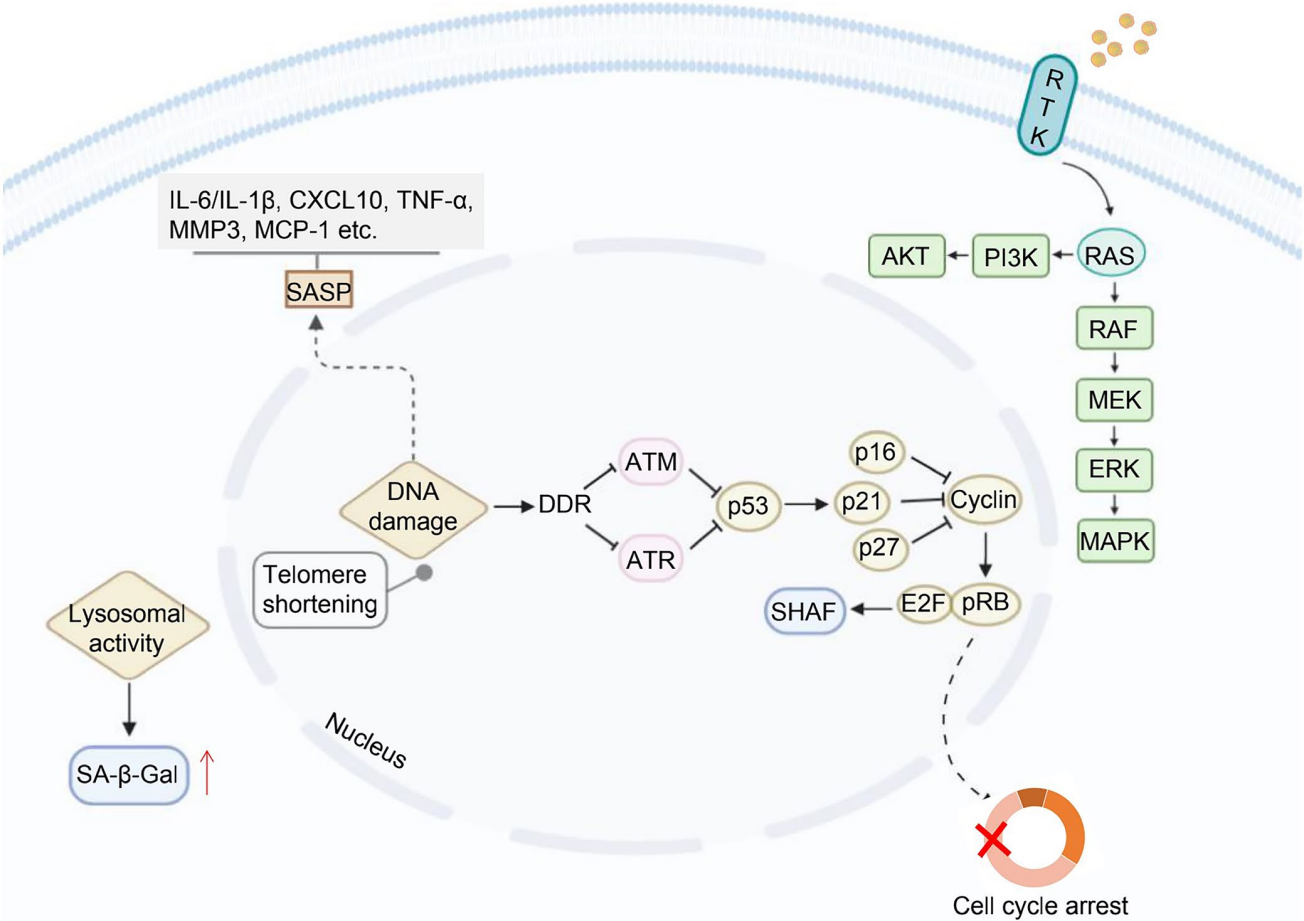
The mechanism of active components promoting the senescence resistance to cancer

These researches clearly showed that the anti‐tumour activities of TCM and the active components of natural products in the form of pro‐senescence can be achieved by influencing the activity of telomerase, DNA damage and activation of oncogenes (Table 1). Meanwhile, the mechanisms involved in these phenomena are summarized (Figure 6).

**TABLE 1:** TCM or active ingredients treatment for malignant tumours

Function	TCM or active ingredients	Classification	Cell Models	Animals Models	Target/Mechanism	Reference
Inhibition of telomerase activity	6‐paradol, 6‐shogaol	Phenolic	A549	Wistar rats	Telomerase activity↓ hTERT expression↓	17
	Pterostilbene	Polyphenols	H460, H1299	/	Telomerase activity↓ hTERT expression↓	20
	Triptolide	Terpenoids	HepG2	BALB/C nude mice	Regulate the AKT and hTERT signalling pathways	28
	Tianshengyuan‐1	/	HL60, CD34+	/	Telomerase activity↓	40
	Baicalein, baicalin	Phenolic flavonoids	HCT116, SW480, HT29, HFF	NSG immunodeficient mice	Regulate MAPK ERK and p38 signalling pathways	25
	Paclitaxel	Terpenoids alkaloids	MEFs	mTREC‐/‐p53‐/‐mice	Telomere dysfunction↓	32
	Papaverine	Alkaloids	HepG2	/	Telomerase activity↓ hTERT mRNA↓	35
Induce DNA damage	Harmine	Alkaloids	MCF‐7	/	hTERT mRNA↓	37
	Icaritin	Flavonoids	HepG2, Huh7	/	ROS↑, DDR↑	45
	Berberine	Alkaloids	A549, HL60, BJ, Siha	/	DDR↑	48
	Hinokitiol	Monoterpenes	A549, H1975, H1299, H3255	NOD‐SCID mice	p53‐independent, DDR↑	50
	Curcumin	Polyphenols	HCT116, MCF7, VSMCs	/	DDR↑	57
	Acetyl‐11‐keto‐b‐boswellic acid (AKBA)	Pentacyclic triterpenoid acid	HepG2, SMMC7721	BALB/C nude mice	DDR↑	53
Oncogene‐induced senescence	Gallotannin	Tannic acid	HCT116	/	ROS↑, DDR↑	55
	Bufalin	Steroid diene	DU145, PC‐3, LNCaP	NSG SCID mice	p53‐dependent	65
	Baicalin	Flavonoids	HCT116, SW480, A549, Panc‐1	BALB/c nude mice	Regulate Ras/Raf/MEK/ERK and p16/Rb signalling pathways	67
SASP	Avenanthramide A	Alkaloids	HEK293T, HCT8, HCT116	C57BL/6J mice	Regulate miR‐129‐3p/Pirh2/p53 signalling pathway	70
	Punicalagin	Polyphenols	BCPAP	/	Regulate NF‐κB signalling pathway	78
	Marchantin C	Bisbibenzyls	A549, H460, H446, H1688	C57BL/6 mice, BALB/c nude mice	p21‐dependent; Regulate NF‐κB signalling pathway	81
Others	1, 8‐cineole	Monoterpenes	HepG2	/	ROS↑	82
	Artemisinin	Terpenoids	A549, NCI‐H1299	/	/	84
	Gypenoside L	Saponins	HepG2, ECA‐109	/	Regulate p38 and ERK MAPK and NF‐ κB pathways	85
	Magnolin	Lignans	TOV‐112D, SKOV3, AsPC‐1, BxPC3, Capan1, Capan2, MIA PaCa‐2, SW480, HCT116, HT‐29, COLO205, MCF7, MDA‐MB‐231, SKBR3, BT‐474	Athymic nude mice	Inhibit the ERKs/RSK2 signalling pathway	86

## 3 DISCUSSION

Recently, TCM and its natural active ingredients have been inclusively explored for the anti‐cancer properties and have been well applied for their advantages of improving the quality of life and prolonging the survival time of patients. They affect the development of malignant tumours in a variety of ways and target different stages of sarcoma, including promoting apoptosis, inhibiting EMT, inducing cycle arrest and promoting senescence, regulating oncogenes and tumour suppressor genes, and interacting with multiple pathways.^87^ Correspondingly, inducing senescence of tumour cells does not cause immediate death, which allows patients to live longer with the tumour. In addition, the dose required to induce senescence of tumour cells is small; therefore, side effects and adverse reactions are reduced to a lower level. Cellular senescence is considered as a potential tumour‐suppressive mechanism, and the induction of cell senescence by active components of TCM and natural product is more likely to be a promising anti‐cancer strategy.

Due to rarely reports on the clinical application of TCM and its natural products in inducing cell senescence against tumour. Therefore, it is of great significance to summarize and analyse the effective components of TCM that have been reported and possess good activity in inducing cell senescence to fight tumour, so as to find new anti‐tumour targets and develop novel anti‐tumour drugs. In this paper, the mechanism of regulating cell senescence in the treatment of malignant tumour by the active components of TCM was also discussed. According to the research, the mechanisms of these active components in regulating cell senescence against malignant tumours involve inhibiting telomerase activity, inducing DNA damage, as well as affecting the microenvironment of tumour cells. However, some active ingredients may be more effective in combination with other drugs due to their limitations in regulating cell senescence against tumours, such as poor water solubility and low bioavailability. For instance, paclitaxel and its water‐soluble conjugates can inhibit tumour development by inducing extensive telomere erosion and triggering cellular senescence.^31^ Interestingly, some of the active ingredients in TCM, such as 1, 8‐cineole, promote cell senescence by blocking the cell cycle and inducing oxidative stress.^82^ However, further in vivo trials and clinical evaluation of these active ingredients are necessary. Meanwhile, it also has reference significance for the research of potential anti‐tumour active drugs. These anti‐tumour molecular mechanisms encourage more researches to focus on especially the exploration of the active ingredients of TCM and natural medicines, to treat various malignant tumours without damaging the surrounding tissues and minimizing the toxic side effects, whereas there are still many problems in applying this treatment to clinical practice. For example, germ cells and hematopoietic stem cells can express certain levels of telomerase activity, and telomerase inhibitors may have adverse effects on these tissues. It is also a tricky problem that cancer reappears on account of a few tumour cells escaping the direct drug‐induced senescence effect. Chronic senescence‐induced inflammation can lead to systemic immunosuppression, and whether this leads to diseases, including cancer, remains an open question. Whether this chronic inflammation causes senescence‐related tissue damage and degeneration remains to be further investigated. Since most studies are confined to a limited number of cancer cell lines in vitro studies, further in vivo evaluation and clinical trials are needed to verify its reliability. It is expected that tumour cells senescence induced by the active ingredients of TCM or natural medicine can be applied in clinical practice, and become a new method for the treatment of malignant tumours, making greater contribution to the improvement of people's happiness index and human health.

## CONFLICT OF INTEREST

No potential conflicts of interest are disclosed.

## AUTHOR CONTRIBUTIONS

HY contributed to conception and design. YL searched the literature. YL, SY and HY wrote the manuscript. KW, JL, XB, RW, YQ, TW and HY critically viewed, edited and approved the manuscript. All authors read and approved the final manuscript.

## Data Availability

Data sharing is not applicable to this article as no new data were created or analysed in this study.
